# Enhanced Intracellular Delivery of BCG Cell Wall Skeleton into Bladder Cancer Cells Using Liposomes Functionalized with Folic Acid and Pep-1 Peptide

**DOI:** 10.3390/pharmaceutics11120652

**Published:** 2019-12-04

**Authors:** Ho Yub Yoon, Hee Mang Yang, Chang Hyun Kim, Yoon Tae Goo, Gwang Yong Hwang, In Ho Chang, Young Mi Whang, Young Wook Choi

**Affiliations:** 1 Drug Delivery Research Lab, College of Pharmacy, Chung-Ang University, 84, Heukseok-ro, Dongjak-gu, Seoul 06974, Korea; phantomryda@naver.com (H.Y.Y.); hope5357@naver.com (H.M.Y.); yj.ch.kim@gmail.com (C.H.K.); rndbsxo5318@naver.com (Y.T.G.); 2 Department of Urology, College of Medicine, Chung-Ang University, 84, Heukseok-ro, Dongjak-gu, Seoul 06974, Koreacaucih@cau.ac.kr (I.H.C.); 3 Department of Internal Medicine, Seoul National University Hospital 101, Daehak-ro, Jongno-gu, Seoul 03080, Korea; ymwhang@gmail.com

**Keywords:** bacillus Calmette–Guérin, cell wall skeleton, liposome, ligand, folate, cell-penetrating peptide, targeted delivery, bladder cancer

## Abstract

Although bacillus Calmette–Guérin cell wall skeleton (BCG-CWS) might function as a potential substitute for live BCG, its use in the treatment of bladder cancer remains limited owing to issues such as insolubility and micrometer-size following exposure to an aqueous environment. Thus, to develop a novel nanoparticulate system for efficient BCG-CWS delivery, liposomal encapsulation was carried out using a modified emulsification-solvent evaporation method (targets: Size, <200 nm; encapsulation efficiency, ~60%). Further, the liposomal surface was functionalized with specific ligands, folic acid (FA), and Pep-1 peptide (Pep1), as targeting and cell-penetrating moieties, respectively. Functionalized liposomes greatly increased the intracellular uptake of BCG-CWS in the bladder cancer cell lines, 5637 and MBT2. The immunoactivity was verified through elevated cytokine production and a THP-1 migration assay. In vivo antitumor efficacy revealed that the BCG-CWS-loaded liposomes effectively inhibited tumor growth in mice bearing MBT2 tumors. Dual ligand-functionalized liposome was also superior to single ligand-functionalized liposomes. Immunohistochemistry supported the enhanced antitumor effect of BCG-CWS, with IL-6 production and CD4 infiltration. Thus, we conclude that FA- and Pep1-modified liposomes encapsulating BCG-CWS might be a good candidate for bladder cancer treatment with high target selectivity.

## 1. Introduction

Bladder cancer is the most common malignancy of the urinary tract and results from the uncontrolled growth of cells that line the bladder wall. Bladder cancer is common in both men and women, with approximately 75% of patients presenting non-muscle invasive bladder cancer at the time of diagnosis [1]. Although this type of bladder cancer can be surgically treated by transurethral resection of the tumor, enabling a high survival rate (88–98%), extensive follow-up is still required as surgical resection is associated with a high recurrence rate of up to 70% [2]. The standard therapy for bladder cancer is a combination of transurethral resection and intravesical instillation of chemotherapeutic agents or immunotherapy with bacillus Calmette–Guérin (BCG), a live attenuated substrain of *Mycobacterium bovis*.

Following its first use by Morales et al. in 1976 [3], BCG has served as the first-line and most effective treatment for bladder cancer. Despite its unique characteristic as cancer immunotherapy that is available in the clinic, BCG has some limitations; it causes severe adverse effects such as BCG abscess, sepsis, and mild cystitis, which affect up to 90% of patients. Furthermore, approximately one-fifth of patients cannot tolerate this therapy [3]. A recombinant BCG has, thus, been developed to reduce the side effects of BCG and induce a more potent therapeutic effect [4]. BCG cell wall skeleton (BCG-CWS), a subunit derived from BCG, is an insoluble fraction of the cell wall consisting of mycolic acids and neutral sugars such as arabinose, galactose, and peptidoglycans. BCG-CWS has been introduced as a highly effective immune activator for bladder cancer immunotherapy [5].

Although the effectiveness of BCG-CWS for cancer immunotherapy has been revealed in several clinical trials [6], limitations still exist regarding its dosage formulation. BCG-CWS is insoluble in both aqueous and organic solvents and reveals a wide size range of 4.7 to 67.8 μm when dispersed in hydrophilic solvents [6]. As a result, an oil-in-water emulsion of BCG-CWS is normally used for human application; however, the use of detergents could induce local irritation and/or inflammation [7]. To efficiently encapsulate BCG-CWS, Nakamura et al. recently derived a unique nanoparticulate technology called liposome evaporated via an emulsified lipid (LEEL) method by using a specific organic solvent that promotes the formation of a nano-sized formulation, with the presence of a condensed structure in the center of the lipid vesicle. With this structure, a compact-shaped of BCG-CWS is established in the center and is covered with lipid bilayers [6].

The liposomal drug delivery system has been widely used to achieve intracellular delivery of various anti-cancer agents, owing to several advantages, including feasible encapsulation of both hydrophilic and lipophilic drugs and excellent biocompatibility and/or biodegradability [8]. However, its lack of selectivity to specific cancer cells continues to serve as a barrier for tumor targeting [9]. To resolve this drawback, liposomes can be surface-modified with different ligands, including peptides, antibodies, small molecules, and carbohydrates [8]. As folate receptors (FRs) are strongly upregulated in different tumors [10], folic acid (FA) is an attractive ligand for targeted delivery. Further, for enhanced intracellular delivery, cell-penetrating peptides (CPPs) have been widely applied [11]. These peptides are relatively short with a net positive charge that enables translocation and successful delivery of various drugs into cells. Pep-1 peptide (Pep1) is one of the most common CPPs with high efficiency of intracellular delivery and a lack of toxicity. Previously, we reported functionalized liposomal systems using these ligands to achieve target cell specificity and improved intracellular uptake of anticancer drug molecules [12].

To develop a novel nanoparticulate system for the efficient delivery of BCG-CWS, we encapsulated liposomes using a modified LEEL method and performed surface functionalization with FA and Pep1. The intracellular uptake efficiency of BCG-CWS-loaded liposomes was evaluated in FR-expressing cancer cell lines, human-derived 5637, and mouse-derived MBT2 bladder cancer cells. The in vitro immunotherapeutic activity was verified by elevated cytokine production and THP-1 migration assay. Using a xenograft mouse model, the antitumor efficacy was demonstrated via tumor growth inhibition and immune cell infiltration into the tumor tissue. Based on the present study, we propose that FA- and Pep1-modified liposomes encapsulating BCG-CWS might be useful for bladder cancer treatment and may display high target selectivity.

## 2. Materials and Methods

### 2.1. Materials

BCG-CWS was sourced from Chungnam National University, Daejeon, Korea. Soy phosphatidylcholine (SPC; purity >99%), 1,2-distearoyl-*sn*-glycero-3-phosphoethanolamine-*N*-[maleimide(polyethylene glycol 2000)] (DSPE-PEG2000-Mal; DP_2K_M), and distearoylphosphatidylethanolamine-polyethylene glycol5000-folate (DSPE-PEG5000-Fol; DP_5K_F) were purchased from Avanti^®^ Polar Lipids (Alabaster, AL, USA). 1,1′-Dioctadecyl-3,3,3′,3′-tetramethylindocarbocyanine perchlorate (DiI), poly-l-lysine (PLL), crystal violet, phosphate-buffered saline (PBS) tablets, and cholesterol (CH) were purchased from Sigma-Aldrich (St. Louis, MO, USA). FA was purchased from Duksan Pure Chemical Co., Ltd. (Seoul, Korea). Pep1 (KETWWETWWTEWSQPKKKRKVC, 22mer) was synthesized by Peptron Co. (Daejeon, Korea). Anti-CD4 antibody and anti-interleukin 6 (IL-6) antibody were purchased from Abcam (Cambridge, UK). Acetonitrile, chloroform, methylene chloride, and other solvents purchased from commercial sources were of cell culture or analytical grade. MBT2 and 5637 bladder cancer cell lines were purchased from the Korean Cell Line Bank (Seoul, Korea). Cell culture materials, including fetal bovine serum, Roswell Memorial Institute (RPMI) 1640 medium, trypsin-ethylenediaminetetraacetic acid, and penicillin-streptomycin, and PBS (10×, pH 7.4) were obtained from Invitrogen (Carlsbad, CA, USA). C3H/HeN mice (6–8 weeks, 20 ± 2 g) were purchased from the Hanlim experimental animal laboratory (Gyeonggi-do, Korea).

### 2.2. Preparation of the Different Liposomal Samples

The following 4 types of BCG-CWS-loaded liposomal nanoparticles were prepared: Plain liposomes (CWS-L), FA-modified liposomes (CWS-FL), Pep1-modified liposomes (CWS-PL), and FA- and Pep1-modified liposomes (CWS-FPL). Based on an earlier report [6], a slightly modified LEEL method was employed to encapsulate BCG-CWS into the liposomes. Briefly, BCG-CWS (3 mg) was dissolved in methylene chloride (900 μL), and the organic solution was emulsified with PBS solution (2100 μL) containing liposomal vesicles (6.45 mM phospholipid equivalent) using a sonicator (Sonoplus, HD 2070; Bandelin Electronics, Berlin, Germany). After removing the solvent by rotary evaporation, extrusion was performed with a mini-Extruder (Avanti^®^ Polar Lipids) using 10 passes through a 200 nm membrane filter. DiI, a red fluorescent probe, was co-encapsulated on purpose for visualizing the cell uptake behavior. Empty liposomes were separately prepared by excluding BCG-CWS or DiI. All liposomal samples were stored in a refrigerator (4 °C) and used in experiments within 3 weeks.

#### 2.2.1. CWS-L

The thin film hydration method was used to prepare the plain liposomal vesicles composed of SPC and CH (9:1 molar ratio). Briefly, the components were dissolved in a methanol-chloroform mixture (2:1 *v/v*), and the organic solvent was rotary evaporated at 40 °C under reduced pressure. The dried thin film was further exposed to a nitrogen gas stream for 2 h and hydrated with 10 mM PBS. The liposomal solution was emulsified with BCG-CWS-containing methylene chloride solution and then extruded as described above.

#### 2.2.2. CWS-FL

To prepare CWS-FL composed of SPC, CH, and DP_5K_F (89.5:10:0.5 molar ratio), the post-insertion method was used as reported previously [13]. Briefly, the prepared CWS-L was incubated with micellized DP_5K_F at 60 °C for 1 h. To prepare micelles, the DP_5K_F solution (1 mg/mL in chloroform) was rotary evaporated, and the dried film was hydrated with PBS solution. The liposomal solution was emulsified with BCG-CWS-containing methylene chloride solution and extruded as described above. Unincorporated DP_5K_F was removed by dialysis for 48 h against distilled water using a cellulose ester dialysis membrane (MWCO 50,000).

#### 2.2.3. CWS-PL

To prepare CWS-PL composed of SPC, CH, DP_2K_M, and Pep1 (89.5:10:0.1:0.05 molar ratio), the post-derivatization method was used as reported earlier [9]. Briefly, maleimide-derivatized liposomes were prepared with DP_2K_M and the lipid composition of CWS-L in PBS solution, emulsified with BCG-CWS-containing methylene chloride solution and extruded as described above. Subsequently, the Pep1 solution in PBS was added to modify the vesicle surface via a thiol-maleimide reaction and allowed to react for 12 h. Unconjugated free Pep1 was removed by dialysis, as described above.

#### 2.2.4. CWS-FPL

CWS-FPL, composed of SPC, CH, DP_5K_F, DP_2K_M, and Pep1 (89.5:9.9:0.5:0.1:0.05 molar ratio), was prepared by combining post-insertion and post-derivatization methods as described above: A 3-step process by preparing maleimide-derivatized liposomes containing BCG-CWS, followed by Pep1 conjugation and post-insertion of the DP_5K_F micelle. Purification was performed by dialysis, as described above.

### 2.3. Particle Size and Zeta Potential (ZP) Analysis

To provide an adequate scattering intensity, liposomal samples were vortexed gently and diluted 1:100 in distilled water before the measurement. Particle size, ZP, and polydispersity index (PDI) were measured in triplicate using a dynamic light scattering particle size analyzer (Zetasizer Nano-ZS; Malvern Instruments, Worcestershire, UK). The particle size of naked BCG-CWS was determined in each sample, and BCG-CWS was dispersed in different organic solvents using a vortex mixer (IKA^®^ vortex GENIUS 3, Staufen, Germany).

### 2.4. Entrapment Efficiency (EE) and Drug Loading (DL)

The EE and DL of BCG-CWS were determined using a previously reported method [6]. Briefly, the liposomes were disrupted with ethanol and centrifuged at 3000 g for 5 min under conditions of 15 °C to obtain the BCG-CWS precipitate. The pellet was dissolved in hexane and mixed with a 0.55% carbol-fuchsin solution. The hexane fraction was then collected, and absorbance at 530 nm was measured using a microplate reader (FlexStation 3; Molecular Devices, Sunnyvale, CA, USA). Liposomal samples were subjected to ultra-filtration using Amicon^®^ ultra-centrifugal filters (MWCO 100,000). To determine the concentration of DiI in the filtrate, an aliquot of the suspension was added to the sample reservoir and centrifuged (14,000 g; 15 °C) for 15 min. For the quantitative determination of DiI, a high-performance liquid chromatography (HPLC) system (Waters^®^ Corporation; Milford, MA, USA) equipped with separation modules (Waters^®^ e2695, Waters^®^ Corporation, Milford, MA, USA), a data station (Empower^®^ 3, Waters^®^ Corporation, Milford, MA, USA), and a fluorescence detector (Waters^®^ W2475, Waters^®^ Corporation, Milford, MA, USA) was used. Using a mobile phase consisted of 0.05 M dimethyl sulfate and methanol (2:98, *v/v*), the chromatography was carried out on a C18 Column (Kromasil^®^, 5 μm, 4.6 × 250 mm; Akzo Nobel, Bohus, Sweden) with the excitation and emission wavelengths of 549 and 565 nm, respectively. The EE and DL were calculated using the following Equations (1) and (2):

(1) $$\mathrm{EE}(\%)=\frac{W_{T}-W_{F}}{W_{T}}\times 100$$

(2) $$\mathrm{DL}(\mu g/\mathrm{mg})=\frac{W_{T}-W_{F}}{W_{L}}$$

where *W_T_*, *W_F_, and W_L_* represent the total amount of the drug (BCG-CWS or DiI) added, the amount of free drug, and the total amount of lipid initially added, respectively.

### 2.5. Conformational Characterization of Ligand Modification

The extent of ligand modification was determined by HPLC assay using a previously reported method [9,14]. Briefly, in the case of the FA ligand, CWS-FL and CWS-FPL were disrupted with 10% Triton X-100, and the content of DP_5K_F was determined using a mobile phase consisting of methanol and 10 mM sodium phosphate buffer (pH 7.0; 92:8, *v/v*) with a C18 column. The eluent was monitored at 285 nm, and the peak for DP_5K_F was separated with the retention time of 2.1 min. Separately, the amount of Pep1 ligand was indirectly quantified by determining the amount of unconjugated Pep1 by HPLC, using a C18 column and a mobile phase consisting of 0.05% trifluoroacetic acid in water (eluent A) and 0.05% trifluoroacetic acid in acetonitrile (eluent B). The eluent gradient ramped from 10% to 60% B in 50 min and subsequently back to 10% B over 5 min. The eluent was monitored at 220 nm, and the peak for Pep1 was separated with the retention time of 32 min.

### 2.6. Transmission Electron Microscopy (TEM)

For morphology examination, liposomes were imaged using a transmission electron microscope (JEM1010; JEOL, Tokyo, Japan) at an acceleration voltage of 80 kV. Briefly, liposomal samples were diluted 100-fold with distilled water and placed on a carbon film grid. The samples were stained with 2% phosphotungstic acid, washed twice with distilled water, and dried at 25 °C prior to observation.

### 2.7. Cell Culture

The 5637 and MBT2 bladder cancer cell lines were incubated in a humidified CO_2_ chamber (Thermo Scientific, Waltham, MA, USA) using RPMI 1640 medium supplemented with penicillin/streptomycin and 10% fetal bovine serum (GIBCO-BRL, Gaithersburg, MD, USA). The cells were sub-cultured every 3–5 days, and cells at passages of 5–20 were used for the experiments.

### 2.8. Flow Cytometry

Intracellular uptake of the liposomes was determined by measuring the mean fluorescence intensity (MFI) of DiI in the liposome by flow cytometry (FACSCalibur; Becton Dickinson, Franklin Lakes, NJ, USA). Briefly, cells were seeded at a density of 3 × 10^5^ cells per well in a 6-well plate with culture media. After 24 h of incubation, the cells were washed twice with PBS and incubated with culture media containing different DiI-loaded liposomes (DiI concentration equivalent to 100 ng/mL). Subsequently, the cells were washed twice with PBS to remove traces of liposomal vesicles remaining within the wells, detached using trypsin-ethylenediaminetetraacetic acid, and suspended in 1 mL of PBS. The suspended cells were introduced into a flow cytometer equipped with a 488 nm argon ion laser. A total of 1 × 10^4^ designated cells were collected to quantify the MFI value. To examine the role of FR binding on liposomal uptake, a competitive binding assay was performed with CWS-FL and CWS-FPL, as previously reported [9,15]. Briefly, 1 mM of free folic acid was added to the culture media 1 h before treatment. After incubation for 2 h at 37 °C, cells were washed twice with PBS to remove unbound liposomes and excess folic acid. The following steps were performed using the same procedure described above. To prove that the positively-charged Pep1 enhances intracellular delivery, PLL (800 μg/mL), an electrostatic interaction inhibitor [16], was pre-incubated for 30 min.

### 2.9. Confocal Laser Scanning Microscopy (CLSM)

To observe the intracellular uptake behavior of liposomes, 5 × 10^4^ cells were seeded in chambered glass slides (Thermo Scientific Nunc, Rochester, NY, USA) and incubated for 24 h at 37 °C, then washed twice with PBS, replenished with fresh culture medium containing the various liposomes, with a DiI concentration of 100 ng/mL. Following incubation for 30 min or 2 h, the cells were washed twice with PBS and fixed with 4% formaldehyde in PBS for 15 min at room temperature. To stain the nuclei and avoid fading, the cells were mounted using Vectashield mounting medium containing DAPI (H-1200). Finally, the cells were observed using a confocal laser scanning microscope (Zeiss LSM 700 Meta; Carl Zeiss Meditec AG, Jena, Germany) under 400× magnification.

### 2.10. Immunoactivity of CWS-Loaded Liposomes

#### 2.10.1. THP-1 Migration

The 5637 cells were seeded in 24-well plates at a density of 5 × 10^4^ cells per well in a culture medium. After 24 h of incubation, cells were treated with different liposomal formulations for 8 h. To the upper chamber of the transwell (Corning, NY, USA), THP-1 cells in serum-free medium (3 × 10^5^) were added and further incubated for 2 h. The cells on the plate and in the lower chamber were subjected to fixation with 4% paraformaldehyde (Biosaesang, Inc., Gyeonggi-do, Korea) and stained with 0.1% crystal violet. Positive THP-1 staining was visualized by an Olympus CKX41 inverted microscope (Olympus, Tokyo, Japan). Three equal-sized fields were randomly selected for THP-1 cell counting, and an average was calculated.

#### 2.10.2. Cytokine Release

The 5637 cells were seeded into a 60-mm cell culture dish at a density of 1×10^6^ cells. After a 24 h incubation, cells were replenished with fresh medium containing the different liposomal formulations. After 72 h of incubation, the culture medium was collected and centrifuged at a speed of 3000 rpm for 10 min at 4 °C. Samples were either immediately analyzed or aliquoted and stored at −80 °C until analysis. Following the manufacturer’s instructions, the absorbance was measured at 540 nm using a microplate reader (FlexStation 3; Molecular Devices, Sunnyvale, CA, USA).

### 2.11. In Vivo Antitumor Efficacy

The animal experiment was approved by the Institutional Animal Care and Use Committee of Chung-Ang University (2019-00061, date: 17 June 2019, Seoul, Korea) and carried out in accordance with the National Institute of Health Guidelines for the Care and Use of Laboratory Animals. Female C3H/HeN mice were randomly divided into 5 groups (*n* = 7): Treatment with the empty liposome (control), CWS-L, CWS-FL, CWS-PL, and CWS-FPL. All mice were subcutaneously inoculated with a mixture of 3.5 × 10^6^ MBT2 cells and BCG-CWS-loaded liposomal formulations (equivalent to 0.1 mg of CWS) via a 21G needle injected into their right flank, except mice in the control group, which were inoculated with a mixture of cells and empty liposomes. A digital caliper (Mitutoyo, Kawasaki, Japan) was used to measure the tumor growth periodically, and tumor volume (mm^3^) was calculated by the formula: (major axis × minor axis^2^) × 0.52 [6]. The change in tumor volume and body weight of each mouse was observed twice per week for 4 weeks. General animal health and potential side effects were monitored in the aspects of impaired movement, behavioral changes, and food or water avoidance. Mice were sacrificed by cervical dislocation at the end of the experiment, and their tumors were excised and weighed. Median survival time was calculated, and Kaplan-Meier survival curves were plotted using GraphPad Prism (GraphPad Software, San Diego, CA, USA). For immunohistochemistry (IHC) analysis, tumors were further fixed with 4% paraformaldehyde. After embedding in OCT compound (Tissue-Tek^®^, Naperville, IL, USA), 3 μm tissue sections were prepared using a cryocut microtome (Leica, Nussloch, Germany).

### 2.12. Statistical Analysis

All values were expressed as the mean ± standard deviation (SD) (*n* ≥ 3). Statistical significance was determined using the Student’s *t*-test, and differences were considered significant at *p* < 0.05.

## 3. Results

### 3.1. Characterization of Liposomes

The compositions and physical characteristics of different liposomal samples are listed in Table 1. Although the particle sizes of ligand-modified liposomes (CWS-FL, CWS-PL, and CWS-FPL) were slightly increased relative to those of CWS-L because of the increased hydrodynamic diameter [17], the average sizes of liposomes ranged from 183 to 189 nm. Regardless of the different compositions, all formulations had PDI values below 0.3, indicating a homogenous nano-dispersion. Based on ZP, the plain liposomes (CWS-L) were negatively charged (−8.3 mV), but because of functional modification, values were changed according to the ligand moiety. FA increased the negative value owing to the anionic effect of the molecule, resulting in −14.3 mV and −12.1 mV for CWS-FL and CWS-FPL, respectively. Conversely, Pep1 induced a charge reversal due to the arginine-based cationic effect, revealing a value of 12.2 mV for CWS-PL. All liposomes had an EE of ~60%. DL ranged from 210.75 to 224.80 μg/mg, thereby displaying a slight variation between the formulations. The EE and DL were not influenced by the addition of DiI, and co-loading with DiI did not affect the physical characteristics of the liposomal samples. In fact, size distribution and ZP were within a similar range (data not shown), while EE and DL of DiI, on average, were 72% and 52 μg/mg, respectively. Such findings revealed that no difference existed between the liposomal formulations (Table S1). Meanwhile, the conformational features were characterized by determining the number of FA and Pep1 molecules located at the liposomal surface, based on the earlier reports [9,12,14]. The total number of lipid molecules that formed a vesicle was estimated by the following formula: 4 π*r*^2 ×^ 2/A, where r and A refer to the radius of the liposome and the cross-sectional area of the SPC head group (0.72 nm^2^), respectively, resulting in 305,000 lipid molecules per vesicle. By accounting the amount of added DP_5K_F (1.8 × 10^19^ molecules) and 50% post-insertion efficiency determined by direct ligand assay, the number of FA ligands was calculated as 709.3 ± 11 per vesicle for both CWS-FL and CWS-FPL. Previously, we found that approximately 51% of added DP_2K_M oriented externally at the liposomal surface [9]. With this assumption, it was possible to estimate the number of externally oriented maleimide groups as approximately 168 molecules per vesicle, which were available for Pep1 conjugation via thiol-maleimide reaction. Pep1 conjugation capability was obtained as approximately 80% from indirect ligand assay. Considering the amount of initially added Pep1 (1.8 × 10^18^ molecules), the number of Pep1 ligands was calculated as 113.5 ± 19 per vesicle for both CWS-PL and CWS-FPL.

**Figure 1:**
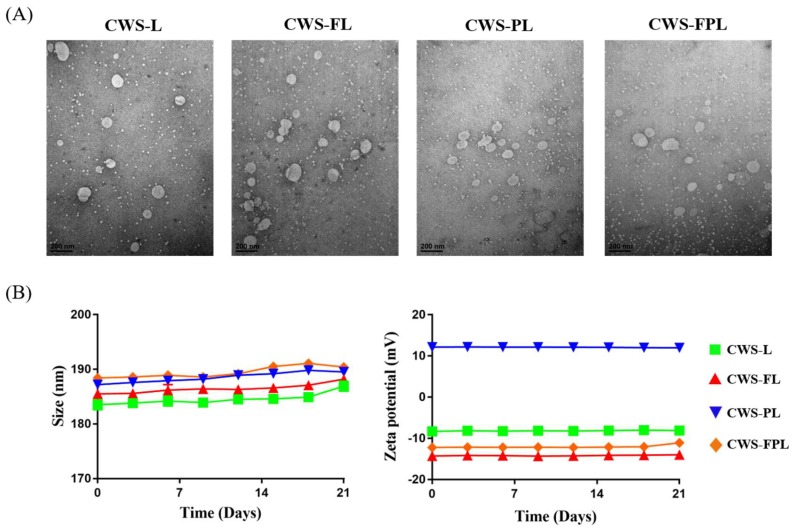
Characterization of the prepared liposomes. (**A**) Transmission electron microscopy (TEM) images of bacillus Calmette–Guérin cell wall skeleton (BCG-CWS)-loaded liposomes. Scale bar indicates 200 nm. (**B**) Stability of the prepared liposomes stored for 3 weeks at 4 °C. Data represent the mean ± standard deviation (SD; *n* = 3).

TEM images revealed no differences between the liposomal samples and demonstrated that the liposome particles were less than 200 nm (Figure 1A). The obtained sizes were, however, slightly smaller than the diameters determined by dynamic light scattering. Dynamic light scattering reflects particle size in its swollen state, whereas TEM reflects it in the dried state [18], and these differences may be responsible for the discrepancy between the results. Figure 1B shows the colloidal stability of various liposomes for 3 weeks. The liposomes were free of any aggregation and displayed uniform size and ZP. Such findings indicated the excellent physical and colloidal stabilities obtained during the experimental period.

### 3.2. In Vitro Cellular Uptake

**Figure 2:**
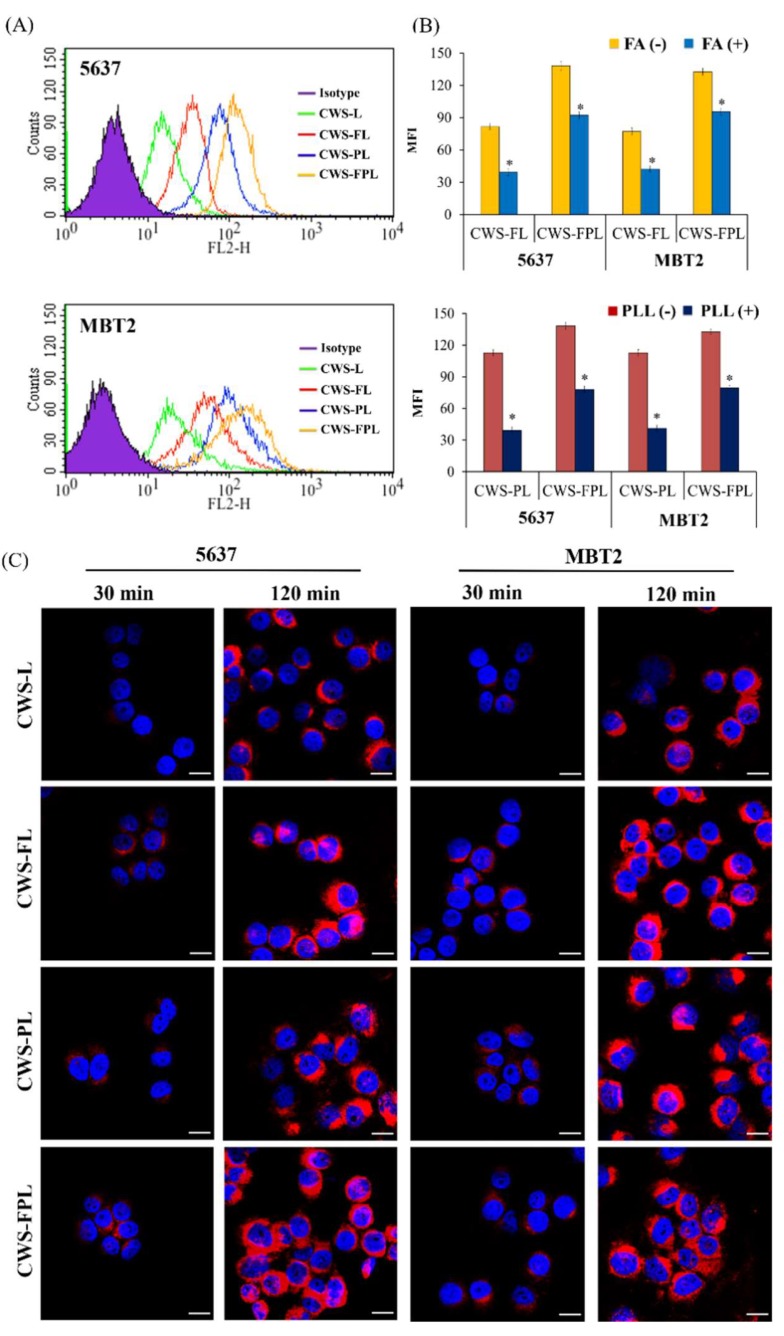
Cellular uptake of different liposomes in the 5637 and MBT2 cell lines. (**A**) Flow cytometry histogram. (**B**) Competitive assay of liposome internalization in the presence (+) or absence (−) of folic acid (FA) and poly-l-lysine (PLL). Data represent the mean ± standard deviation (SD; *n* = 3). * Significantly different at *p* < 0.05. (**C**) Confocal laser scanning microscopy (CLSM) images. Blue and pink fluorescence represents the nucleus and DiI, respectively. Scale bar indicates 100 μm.

The intracellular delivery of different liposomes was evaluated using the 5637 and MBT2 cell lines, which were well-known FR-expressing cancer cells that have been widely used to evaluate FR targeting [19]. As shown in the flow cytometry histogram (Figure 2A), in both cells, the fluorescence peak shifted to the right owing to liposomal treatment compared to no treatment (Isotype). Moreover, the shifting degrees of CWS-FL, CWS-PL, and CWS-FPL were revealed to be greater than those of CWS-L, indicating that the ligand modification remarkably enhanced the cellular uptake. For a quantitative comparison of the histogram shift, MFI was adopted as a parameter. MFI values were observed in the order, CWS-FPL (138.26) > CWS-PL (112.69) > CWS-FL (81.72) > CWS-L (34.95) for 5637 cells; and CWS-FPL (132.59) > CWS-PL (112.58) > CWS-FL (77.14) > CWS-L (35.2) for MBT2 cells. To confirm whether the enhanced intracellular uptake was attributed to ligand modification, a competitive binding assay was performed in the presence or absence of FA and PLL (Figure 2B). The relevant flow cytometry histograms are shown in Figure S1. Through FA-pretreatment in both cell lines, the MFI values of CWS-FL and CWS-FPL were significantly suppressed at *p* < 0.05 compared to that of the untreated cells. In addition, pretreatment with PLL as an electrostatic interaction inhibitor greatly reduced the uptake of Pep1-modified liposomes, CWS-PL, and CWS-FPL, by 2.9-fold and 1.8-fold in 5637 cells, and 2.7-fold and 1.7-fold in MBT2 cells, respectively, compared to the untreated cells. As a result, the role of ligand modification for the enhanced cellular uptake was clearly demonstrated. Based on the suppression level, it was also found that the effect of Pep1 modification was relatively greater than that of FA modification.

The internalization of different liposomes was further visualized by CLSM (Figure 2C). In both cell lines, time-dependent uptake behaviors were found. During the initial 30 min, CWS-L displayed no fluorescence, while the other liposomes revealed a weak response. Although the response of CWS-L was weaker than that of the functionalized liposomes, after 2 h, a strong fluorescence was observed in all liposomes. Unfortunately, the difference between the functionalized liposomes was not clearly distinguishable. However, in 5637 cells, an appreciable superiority of CWS-FPL relative to CWS-FL and CWS-PL was demonstrated, revealing a greater density of scattered dot-like fluorescence throughout the cytoplasm at 2 h.

### 3.3. In Vitro Immunoactivity of the BCG-CWS-Loaded Liposomes

**Figure 3:**
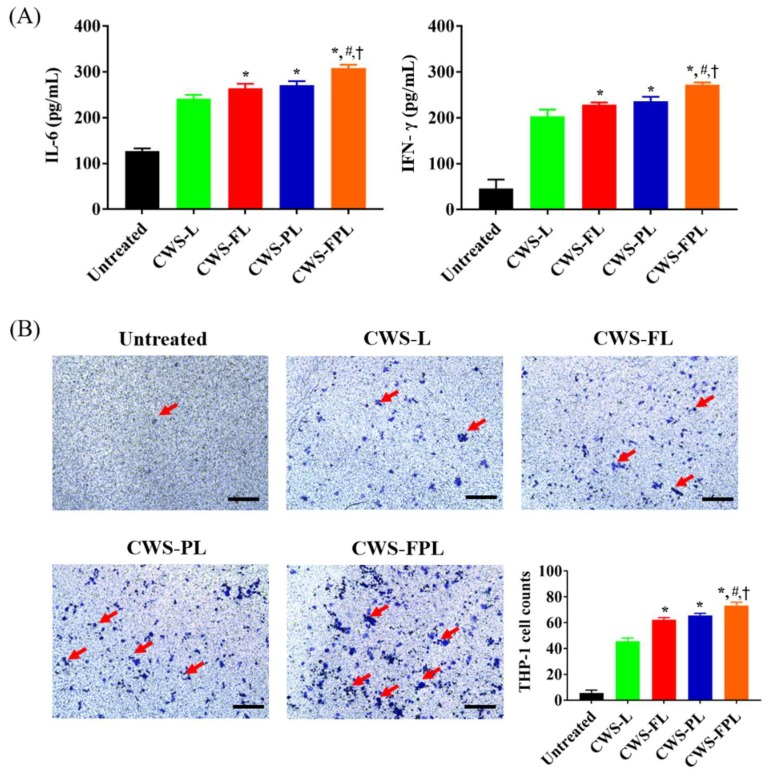
Immunoactivity of the bacillus Calmette–Guérin cell wall skeleton (BCG-CWS)-loaded liposomes in 5637 cells. (**A**) Production of various cytokines. (**B**) Chemotaxis of THP-1 cells. Red arrows indicate cell migration. Scale bar indicates 250 μm. Data represent the mean ± standard deviation (SD; *n* = 3). Significantly different at *p* < 0.05: * versus plain liposomes (CWS-L); ^#^ versus folic acid (FA)-modified liposomes (CWS-FL); ^†^ versus Pep1-modified liposomes (CWS-PL).

The immunoactivity of internalized BCG-CWS was analyzed via cytokine production and a THP-1 migration assay. The mean cytokine levels of different groups following CWS-liposome treatments in the 5637-cell line are shown in Figure 3A. A significant increase in IL-6 was detected in functionalized liposomes compared to CWS-L. However, CWS-FPL displayed the highest values, with the order of CWS-FPL (308.22 ± 7.24) > CWS-PL (270.83 ± 9.14) > CWS-FL (264.21 ± 9.86) > CWS-L (241.60 ± 8.31). The expression of interferon (IFN)-γ was increased in the order of CWS-FPL (272.50 ± 4.56) > CWS-PL (235.71 ± 9.96) > CWS-FL (228.86 ± 4.53) > CWS-L (203.52 ± 14.75). In addition, the effect of CWS-liposomes on chemotaxis was evaluated by the THP-1 migration assay (Figure 3B). All formulations induced a higher migration rate of THP-1 cells, with the order of CWS-FPL (73.33 ± 2.49) > CWS- PL (65.66 ± 1.42) > CWS-FL (62.33 ± 1.57) > CWS-L (45.66 ± 1.87). This result is consistent with the result of cytokine production, confirming the excellent immunoactivity of CWS-FPL.

### 3.4. In Vivo Antitumor Efficacy

**Figure 4:**
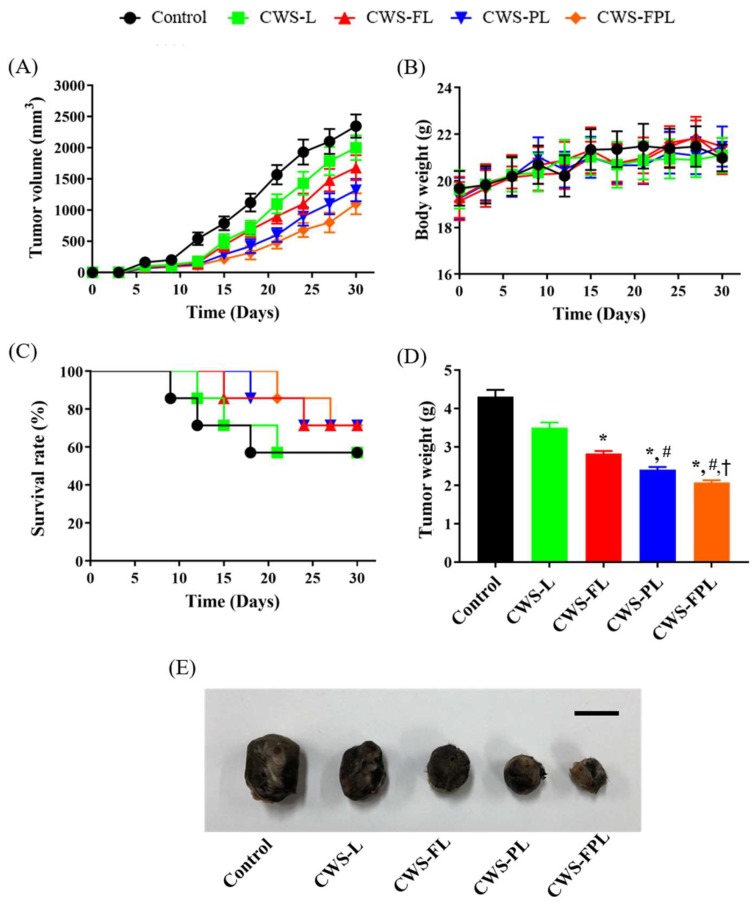
In vivo antitumor efficacies in MBT2 cell-bearing C3H/HeN mice treated with different formulations. (**A**) Changes in tumor volume at one-month post-administration (*n* = 4–7). (**B**) Body weight changes (*n* = 4–5). (**C**) Survival curves. (**D**) Weights of excised tumors at the end of the study (*n* = 4–5). (**E**) Representative images of excised tumors for different treatment groups. Scale bar indicates 10 mm. Significantly different at *p* < 0.05: * versus plain liposomes (CWS-L); ^#^ versus folic acid (FA)-modified liposomes (CWS-FL); ^†^ versus Pep1-modified liposomes (CWS-PL).

The antitumor effects of BCG-CWS-loaded liposomal formulations were evaluated using the MBT2 tumor xenograft models on C3H/HeN mice. As shown in Figure 4A, after 10 days of inoculation, the tumor volume for the control group was rapidly increased relative to that of the liposome-treated groups. At the end of the experiment, tumor volumes reached approximately 2350, 2000, 1680, 1320, and 1100 mm^3^ for the control, CWS-L, CWS-FL, CWS-PL, and CWS-FPL groups, respectively. CWS-L was less effective at controlling tumor growth inhibition compared to the functionalized liposomes, and demonstrated a significant difference after 3 weeks. Compared to the treatment group with single ligand-conjugated liposomes (CWS-FL and CWS-PL), significant tumor growth inhibition was observed for dual ligand-conjugated liposomes (CWS-FPL), indicating the synergistic effect of the dual ligands, FA (targeting), and Pep1 (cell-penetrating).

Figure 4B shows the body weight changes of mice during the experiment. The body weights were monitored to determine the adverse side effects of the different therapeutic regimens. No significant weight changes were identified in any group, indicating the excellent safety of the tested formulations. In general, the body weights of the xenograft mice tended to increase with time owing to inherent body growth and increased tumor volume [20]. Therefore, mice in the present study might have been subjected to “tumor-borne lean body” where their weights gradually decreased with time. Conversely, tumor volume was substantially increased. As a result, with the compensation of tumor mass, overall body weights were not significantly different between treatment groups. Figure 4C depicts the survival curves, where the first death was observed in the control group on day 9, followed by the CWS-L, CWS-FL, and CWS-PL groups on days 12, 15, and 18, respectively. No death occurred in the CWS-FPL group until day 21, suggesting that using CWS-FPL could ensure longer survival time than that by other liposomes.

At the end of the experiment, the excised tumors were weighed and photographed (Figure 4D,E) to perform a further comparison. Tumor weights closely correlated with tumor volume, and CWS-FPL had the least tumor weight of the formulations. Based on these data, the percentage of tumor growth inhibition (TGI) was calculated as follows [21]: TGI (%) = (1 − *W_t_*/*W_c_*) × 100, where *W_t_* and *W_c_* represent the final tumor weight of the treated and control groups, respectively. As listed in Table 2, the degree of TGI was in the order of CWS-FPL > CWS-PL > CWS-FL > CWS-L. As a result, the anti-tumor efficacy of BCG-CWS was improved by the surface modification of liposomes with the specific ligands, FA or Pep1, and further improved with dual ligand-functionalized liposomes (CWS-FPL).

### 3.5. Immunohistological Analysis

**Figure 5:**
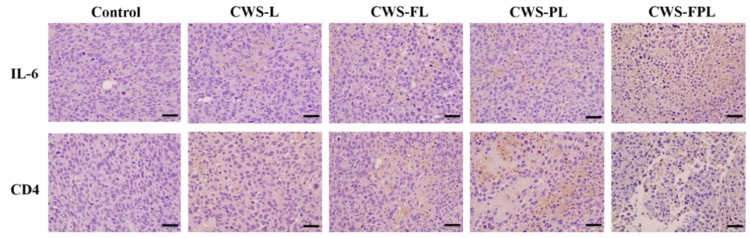
Expression of anti-interleukin 6 (IL-6) and CD4 in MBT2 xenograft tumor tissues treated with the different liposomes. Scale bar indicates 200 nm at 400× magnification.

As the antitumor effect of BCG-CWS was known to be mediated by the activation of the immune system and the induction of an inflammatory response [22], IL-6 production, and CD4 infiltration were evaluated by IHC after tumor tissues were harvested. As shown in Figure 5, the expression level of IL-6 and CD4 (as indicated by brown spots) was only faintly visible in the control group. However, in the liposome-treated groups, brown spots were clearly manifested. In particular, CWS-FPL revealed the most prominent spot changes for both IL-6 and CD4. An immunohistochemical score was used to further compare the immunoreactivity. These scores, which were determined by a specialized pathologist, were graded based on the following criteria: 0 (negative); 1 (minimal); 2 (slight); 3 (moderate); 4 (strong). As listed in Table 2, the IHC scores of the control group were zero for both IL-6 and CD4, while those of the liposome-treated group ranged from 1 to 4. The expression level was in the order of CWS-FPL ≥ CWS-PL > CWS-FL ≥ CWS-L, which was closely related to the TGI results. Altogether, we suggest that the liposome-derived internalization of BCG-CWS into bladder tumor tissues increases IL-6 production and CD4 infiltration, thereby increasing the antitumor efficacy.

## 4. Discussion

After the antitumor activity of BCG-CWS was revealed, many studies, over time, employed this substitute as cancer immunotherapy [23,24,25]. The main immune activator of BCG is derived from its CWS, which elicits an immune response [26]. However, its large size and insolubility in aqueous media serve as drawbacks to its use. BCG-CWS is a very large molecule that contains mycolic acid-arabinogalactan-peptidoglycan. As BCG-CWS must be internalized by the bladder cancer cells to initiate the anti-tumor effect, a suitable formulation is a pre-requisite for its use in bladder cancer treatment. In a previous study, liposomal encapsulation of BCG-CWS was attempted using a conventional hydration method; however, aggregation occurred, which exceeded 1 µm. This aggregation was mainly attributed to the hydrophobic interaction among the mycoloyl moieties [26]. Alternatively, by employing an appropriate organic solvent of pentane, Nakamura et al. developed a “LEEL” method, where the solvent containing BCG-CWS was emulsified with a liposomal solution, followed by solvent evaporation and extrusion. During this encapsulation, BCG-CWS in the hydrophobic solvents becomes smaller and more compact via the formation of a multilayered rolled sheet by inward rolling of its hydrophilic peptidoglycan layer [27], thereby encapsulating BCG-CWS using lipids to hide it from the hydrophilic environment.

The choice of a suitable solvent for BCG-CWS solvation is a critical factor for nano-sized liposomal encapsulation. In a previous report by Nakamura et al. [6], pentane was chosen for BCG-CWS dispersion, resulting in a compact size of 96 ± 1 nm. However, in our preliminary study, the use of pentane raised a problem: Size distribution was not uniform and even fluctuated as time passed, which might be attributed to the volatile property of pentane (boiling point 36.1 °C). Therefore, to find an alternative, the size distribution of BCG-CWS in different solvents was measured (Table S2). As methylene chloride had the smallest and most uniform size and displayed excellent reproducibility among the solvents screened, it was selected as the optimal solvent. The dipole moment of methylene chloride is 1.60, while that of the other solvents is higher, indicating that the size of BCG-CWS depends on the polarity of the solvent used. The preparation of liposomes involved an oil-in-water emulsification and solvent evaporation steps, where the phospholipid functioned as an amphipathic emulsifier. When the solvent was evaporated, BCG-CWS was encapsulated in its hydrophobic form into the liposomes [6]. However, this type of conventional liposome has a limitation for drug targeting owing to its lack of cell specificity. Unfortunately, to date, there are no trials where bladder cancer cells have been targeted using functionalized liposomes encapsulating BCG-CWS.

For decades, CPPs have been widely used for enhanced intracellular drug delivery [28]. In a previous study, we introduced Pep1 into liposomes, which remarkably facilitated the translocation of macromolecules, and FITC-dextran encapsulated liposomes [9]. CPPs might be able to solve the issues related to insufficient intracellular drug delivery. However, because these CPPs are non-specific in their targeting of cancer cells, their application has been limited. To overcome this obstacle, introducing a targeting ligand would be preferred. Among the various types of ligands for surface modification, FA has been extensively used to target FR, which is restrictively expressed in normal cells but highly expressed in various malignant tumors of epithelial origin [29]. Previously, we established a selective and enhanced drug delivery system using FA-tethered and Pep1-modified liposomes [9]. Here, different lengths of PEG linker for ligand modification were used: PEG5000 for FA and PEG2000 for Pep1. With this strategy, the FA ligand can orient the outermost layer, thus efficiently targets FR without steric hindrance. Consequently, the number of ligands was optimized with approximately 700 FA molecules and 110 Pep1 molecules. This was achieved via the addition of folate and peptide at respective ratios of 0.5 and 0.05 mol to the liposomes composed of SPC and CH. Based on this optimized liposomal system, we constructed single ligand-modified liposomes (CWS-FL and CWS-PL) and dual ligand-modified liposomes (CWS-FPL) using FA and/or Pep1 to increase the translocation of BCG-CWS-encapsulating liposomes to bladder cancer cells with specific selectivity.

Physical characteristics such as size, drug entrapment, ZP, and colloidal dispersion stability are crucial for nanocarrier formulation. In the present study, all prepared liposomes were less than 200 nm, revealing no influence of ligand modification on vesicular size. Earlier, it was found that FA or Pep1 modification at low molar ratio did not alter the size of liposomes, because the hydrodynamic diameter of the particles could be mainly dominated by the long PEG chains [9]. With this nano-size, liposomal carriers can readily translocate into the tumoral tissues [30,31]. BCG-CWS was encapsulated into the liposomes, with EE values ranging from 59% to 62%. As these values were similar to those in a previous study [6], the liposomes were deemed to be successfully prepared for BCG-CWS encapsulation. The colloidal stability of the liposomes in the PBS solution was maintained for 3 weeks; thereafter, no signs of aggregation were revealed. Based on ZP, CWS-FL had a higher negative charge than CWS-L, which might be attributed to the presence of negatively charged functional groups derived from the DP_5K_F anchored on the liposome surface [19]. For CWS-PL, the ZP shifted toward a positive charge, which was clearly derived from the positively-charged Pep1. Conversely, CWS-FPL displayed a net negative charge, which might be owing to the difference in PEG chain length: The DP_5K_F anchored with a longer PEG covers the Pep1 ligand anchored via DP_2K_M with a shorter PEG chain.

To achieve a sufficient antitumor effect, BCG-CWS should be translocated into the target cancer cell lines. Therefore, in vitro cellular uptake was carried out to determine the efficiency of liposome internalization in 5637 and MBT2 bladder cancer cells. As shown in Figure 2, the cellular uptake efficiencies of the functionalized liposomes were remarkably higher than that of CWS-L, demonstrating that cellular uptake can be facilitated using the ligands FA and Pep1. Although dual ligand-modified liposomes, such as CWS-FPL, are expected to exhibit enhanced cellular uptake owing to their synergistic effect, their internalization was not drastically higher than that of CWS-FL and CWS-PL. This behavior might be owing to the steric hindrance of the two ligands, as discussed above. To an extent, this result is consistent with the results of earlier studies where the use of high molecular weight PEG was proposed to impede uptake efficiency [32,33]. Nonetheless, these results prove that the ligands on the surface of the liposomes interact with their respective receptors, resulting in enhanced uptake when the intracellular concentration of the therapeutic agents is increased. To further investigate the uptake mechanism of the liposomal nanocarriers for BCG-CWS delivery, a competitive assay was carried out. First, the results of this study owing to FA-pretreatment clearly revealed that the uptake process of CWS-FL and CWS-FPL occurred via receptor-mediated endocytosis. This finding is consistent with numerous results that highlight the efficiency of FR-specific drug targeting. Second, PLL was used to determine the effect of the positively-charged Pep1, which inhibits endocytosis by interacting with the negative charge on the surface of cells [16]. Such findings imply that PLL-pretreatment significantly suppressed the extent of CWS-PL uptake, suggesting that the translocation ability of the CPP-modified liposomes firmly relies on the existence of Pep1. Based on these results, we suggest that the functionalized liposomes could deliver BCG-CWS into the bladder cancer cells with high selectivity and efficient intracellular translocation, thereby enhancing the immunotherapeutic activity.

The immunoactivity of BCG-CWS was assessed by determining cytokine production and THP-1 cell migration after liposomal treatment. Although the exact mechanism whereby BCG exerts its anti-tumor efficacy remains unclear, many studies have shown that the activation of the immune system and induction of an inflammatory response are involved in the antitumor effect [34,35]. The BCG-CWS-mediated antitumor effect has been shown to be similar to that of BCG [22]. The internalization of BCG is known to induce cytokine production by tumor cells, which can be directly toxic to these cells. In addition, BCG interacts with macrophages, enabling the production of cytokines and presentation of BCG-related antigens to T lymphocytes, ultimately resulting in the direct killing of tumor cells [35]. As the massive release of cytokines, including IL-6 and IFN-γ, occurs following BCG therapy, we evaluated the production of these cytokines to determine the immunotherapeutic effect of BCG-CWS. Because bladder cancer cells secrete IL-6 in response to BCG in vitro [36], the obtained results imply that BCG-CWS was successfully translocated into the 5637 cells. IFN-γ is a type of cytokine that plays an important role, including as a direct cytotoxic agent against bladder carcinoma. IFN-γ also stimulates the cytotoxicity of macrophages against bladder cancer cells [37]. Based on our findings, IFN-γ levels were elevated by liposomal treatment. In addition, the monocyte chemotactic effects in the THP-1 migration assay revealed that liposomes increased the migration of THP-1, thereby indicating immune-related cell recruitment. Taken together, the results demonstrate that BCG-CWS encapsulation into liposomes could maintain its inherent immunotherapeutic effect, despite the remarkable difference between the formulations.

**Figure 6:**
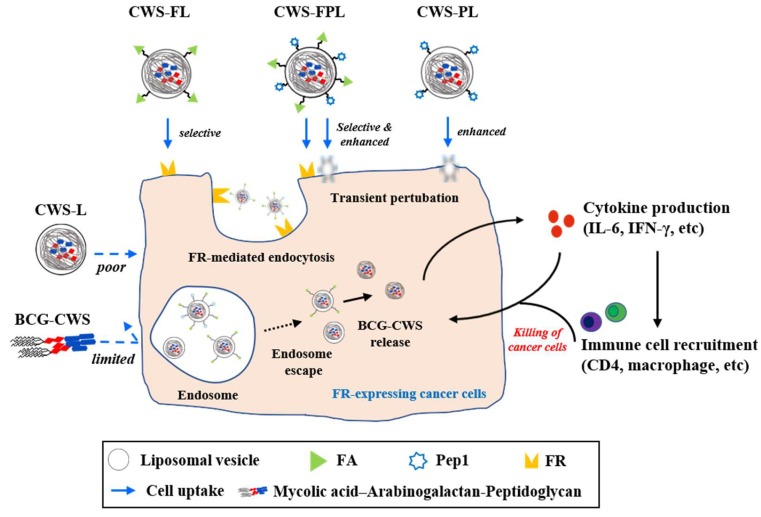
Schematic illustration of the proposed intracellular translocation pathway of different liposomes, endosome escape, bacillus Calmette–Guérin cell wall skeleton (BCG-CWS) release, and the resulting cascade of the immune response. The intracellular uptake behavior varied according to the type of liposomal nanocarrier: The uptake of naked BCG-CWS or plain liposomes (CWS-L) was limited or poor. However, functionalization enabled uptake enhancement via FR-mediated endocytosis (FA-modified) or transient perturbation/penetration (Pep1-modified). The BCG-CWS released into the cytosol triggered a cascade of immune response via cytokine production and immune cell recruitment, thereby exhibiting antitumor efficacy.

In terms of in vivo antitumor efficacy of BCG-CWS, its internalization is expected to result in immune activation, including CD4 infiltration and cytokine production. All BCG-CWS-loaded liposomes caused tumor growth inhibition; however, the degree of suppression was further differentiated by ligand-modified liposomes. As depicted in Figure 6, the observed intracellular uptake behavior was dependent on the type of liposomes used, specifically the type of ligand-modification. Although the uptake of naked BCG-CWS is extremely limited, that of CWS-L is relatively poor. Based on the ligand used, however, functionalized liposomes displayed enhanced uptake via the following strategies: CWS-FL selectively binds to the FR, resulting in FR-mediated endocytosis; CWS-PL is internalized into the cytosol via cell membrane perturbation caused by Pep1, and CWS-FPL displays a considerably enhanced uptake that involves the combined effect of FA and Pep1. Once BCG-CWS is released into the cytosol, different cytokines are produced that can be cytotoxic to tumor cells. Simultaneously, immune cells such as CD4 and macrophage could infiltrate the cancer cells, resulting in their death.

Numerous studies have shown the antitumor effect of BCG-CWS, which triggers a cascade of immune responses [22]. Thus, to better understand its antitumor effect, IL-6 and CD4 were employed. IL-6 is one of the main cytokines released from bladder cancer cells [38], and it recruits neutrophils to the site of the cancer, which also contributes to its anticancer effects [34]. The IHC results revealed that the BCG-CWS-loaded liposomes were internalized by bladder cancer cells. This is because the release of IL-6 is known to be dependent on the internalization of BCG, which might be owing to the BCG-CWS used herein. As brown spots were clearly visible, this implied the production of IL-6. Nonetheless, the difference in formulation was further elucidated according to the immunohistochemical scores. CD4, a type of T lymphocyte, is considered to be one of the most effective immune cells for eliminating cancer cells [39]. In an earlier study, CD4 and CD8 lymphocytes were shown to be essential for successful BCG immunotherapy. This was proven in a murine bladder cancer model where depletion of either CD4 or CD8 lymphocyte resulted in a loss in BCG-mediated antitumor activity [40]. In the present study, we evaluated CD4 expression using the BCG-CWS-loaded liposomes to determine the correlation between the enhanced antitumor effect and CD4 infiltration. As all liposome-treated groups showed brown spots, the difference between the groups was further visualized through an enlargement of the brown spot region. The IHC scores clearly revealed that enhanced internalization of BCG-CWS-loaded liposomes resulted in the elevation of CD4 infiltration. Altogether, these findings indicate that the encapsulation of BCG-CWS into the functionalized liposomes is beneficial for intracellular translocation of the cargo into bladder cancer cells, ultimately mediating the antitumor effect with IL-6 and CD4.

## 5. Conclusions

By employing a modified LEEL method in the present study, we could efficiently encapsulate BCG-CWS into the liposomes. Furthermore, we could successfully functionalize the liposomal surface with the specific ligands, FA and Pep1, which were the respective targeting and cell-penetrating moieties. Based on cell specificity and enhanced internalization, the functionalized liposomes had significant enhancements in their in vitro immunoactivity and in vivo antitumor efficacy. Thus, we concluded that CWS-FPL might be a good candidate system for bladder cancer immunotherapy. However, because this animal study was limited to a xenograft mouse model, further studies using an orthotopic bladder cancer animal model are still needed to demonstrate the intravesical instillation of this immunotherapy.

## Figures and Tables

**Table 1:** Composition and physical properties of the prepared liposomes.

Formulations	CWS-L	CWS-FL	CWS-PL	CWS-FPL
*Liposome composition (mol ratio)*				
SPC	90	89.5	89.9	89.5
CH	10	10	10	9.9
DP_5K_F		0.5		0.5
DP_2K_M			0.1	0.1
Pep1			0.05	0.05
*Physical properties*				
Size (nm)	183.5 ± 0.21	187.5 ± 0.14	189.2 ± 0.23	189.4 ± 0.23
PDI	0.28 ± 0.08	0.21 ± 0.05	0.27 ± 0.05	0.24 ± 0.08
ZP (mV)	−8.31 ± 0.37	−14.29 ± 0.37	12.16 ± 0.25	−12.11 ± 0.22
EE (%)	61.32 ± 0.37	59.89 ± 0.16	60.12 ± 0.33	62.17 ± 0.52
DL (μg/mg)	219.86 ± 3.53	223.49 ± 2.34	224.80 ± 2.77	210.75 ± 3.12

Data represent the mean ± SD (*n* = 3).

**Table 2:** Antitumor efficacy of the different treatment groups in the MBT2 bearing xenograft mouse model.

Treatment Groups	Average Tumor Weight (g)	Tumor Growth Inhibition (%)	IHC Score	
			IL-6	CD4
Control	4.31 ± 0.15	-	0	0
CWS-L	3.51 + 0.12	18.56	2	1
CWS-FL	2.83 ± 0.06	34.38	2	2
CWS-PL	2.42 ± 0.06	43.85	3	4
CWS-FPL	2.26 ± 0.05	47.56	4	4

Data represent mean ± standard deviation (SD; *n* = 4–5).

## Supplementary Materials

The following are available online at https://www.mdpi.com/1999-4923/11/12/652/s1, Table S1: Entrapment efficiency (EE) and drug loading (DL) of 1,1′-dioctadecyl-3,3,3′,3′-tetramethylindocarbocyanine perchlorate (DiI) in bacillus Calmette–Guérin cell wall skeleton (BCG-CWS)-loaded liposomes; Table S2: Size and polydispersity index (PDI) values of bacillus Calmette–Guérin cell wall skeleton (BCG-CWS) in the selected organic solvents. Figure S1: Flow cytometry histograms for the competitive assay of various liposome internalization to the 5637 and MBT2 cell lines in the presence (+) or absence (−) of FA and PLL.
