# Supplementary material for: Enhanced Intracellular Delivery of BCG Cell Wall Skeleton into Bladder Cancer Cells Using Liposomes Functionalized with Folic Acid and Pep-1 Peptide
Source: Pharmaceutics. 2019 Dec 4;11(12):652. doi: 10.3390/pharmaceutics11120652 (PMC6970232; doi:10.3390/pharmaceutics11120652)
Supplement: Supplementary file 1 [file pharmaceutics-11-00652-s001.pdf]

Supplementary Materials

Table S1. Entrapment efficiency (EE) and drug loading (DL) of 1,1′-dioctadecyl-3,3,3′,3′-tetramethylindocarbocyanine perchlorate (DiI) in bacillus Calmette–Guérin cell wall skeleton (BCG-CWS)-loaded liposomes.

Formulation	EE (%)	DL (μg/mg)
CWS-L	72.45± 0.50	53.06± 0.16
CWS-FL	72.29± 0.18	51.63± 1.12
CWS-PL	72.36± 0.16	52.04± 0.49
CWS-FPL	72.29± 0.28	51.62± 0.47

Data represent mean ± SD (n = 3)

Table S2. Size and polydispersity index (PDI) values of bacillus Calmette–Guérin cell wall skeleton (BCG-CWS) in the selected organic solvents.

Solvent	Dipole moment	Size (nm)	PDI
Methylene chloride	1.6	113.73 ± 1.76	0.184 ± 0.01
Tetrahydrofuran	1.75	156.37 ± 4.23	0.21 ± 0.02
Acetone	2.88	287.01 ± 11.68	0.24 ± 0.01
Chloroform	3.44	303.34 ± 24.63	0.321 ± 0.02

Data represent mean ± SD (n = 3)

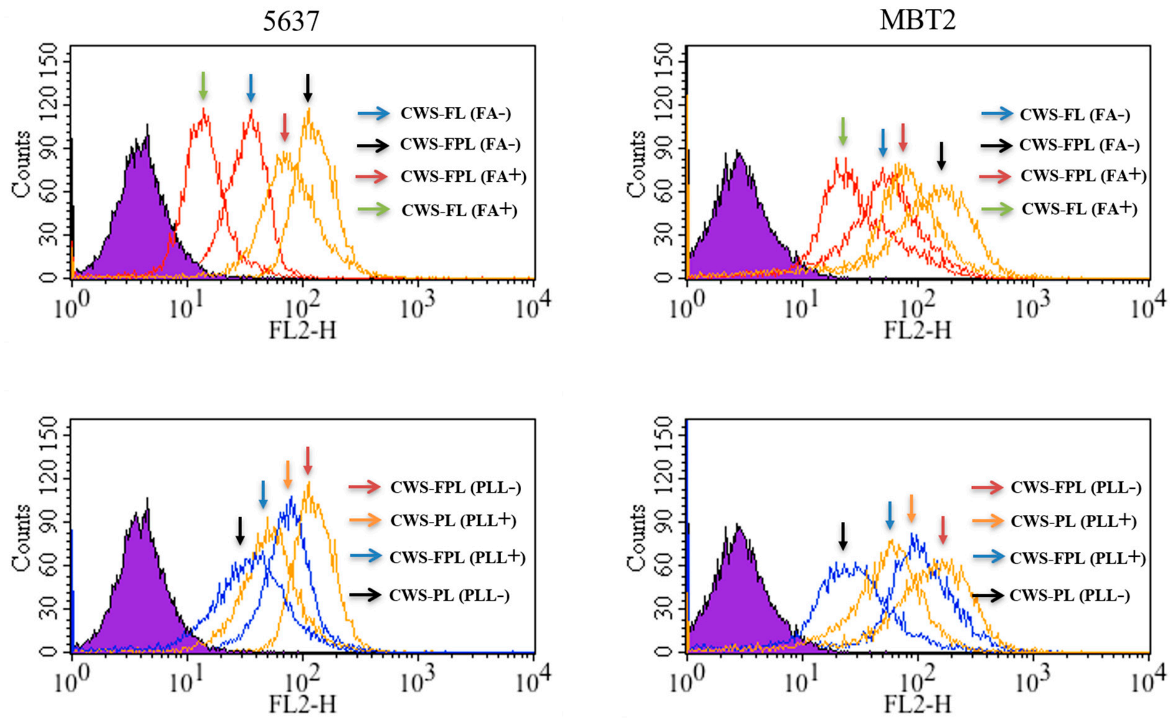

Figure S1. Flow cytometry histograms for the competitive assay of various liposome internalization to the 5637 and MBT2 cell lines in the presence (+) or absence (-) of FA and PLL.
